# Gestational Diabetes Is Characterized by Reduced Mitochondrial Protein Expression and Altered Calcium Signaling Proteins in Skeletal Muscle

**DOI:** 10.1371/journal.pone.0106872

**Published:** 2014-09-12

**Authors:** Kristen E. Boyle, Hyonson Hwang, Rachel C. Janssen, James M. DeVente, Linda A. Barbour, Teri L. Hernandez, Lawrence J. Mandarino, Martha Lappas, Jacob E. Friedman

**Affiliations:** 1 Department of Pediatrics, University of Colorado Denver School of Medicine, Aurora, Colorado, United States of America; 2 Department of Medicine, University of Colorado Denver School of Medicine, Aurora, Colorado, United States of America; 3 Department of Obstetrics and Gynecology, University of Colorado Denver School of Medicine, Aurora, Colorado, United States of America; 4 Boston Children’s Hospital/Harvard Medical School, Boston, Massachusetts, United States of America; 5 Department of Obstetrics & Gynecology, Brody School of Medicine, East Carolina University, Greenville, North Carolina, United States of America; 6 Department of Kinesiology, Arizona State University, Tempe, Arizona, United States of America; 7 Department of Medicine, Mayo Clinic Arizona, Tempe, Arizona, United States of America; 8 Department of Obstetrics and Gynaecology, University of Melbourne, Melbourne, Victoria, Australia; 9 Mercy Perinatal Research Centre, Mercy Hospital for Women, Heidelberg, Victoria, Australia; University of Padova, Italy

## Abstract

The rising prevalence of gestational diabetes mellitus (GDM) affects up to 18% of pregnant women with immediate and long-term metabolic consequences for both mother and infant. Abnormal glucose uptake and lipid oxidation are hallmark features of GDM prompting us to use an exploratory proteomics approach to investigate the cellular mechanisms underlying differences in skeletal muscle metabolism between obese pregnant women with GDM (OGDM) and obese pregnant women with normal glucose tolerance (ONGT). Functional validation was performed in a second cohort of obese OGDM and ONGT pregnant women. Quantitative proteomic analysis in rectus abdominus skeletal muscle tissue collected at delivery revealed reduced protein content of mitochondrial complex I (C-I) subunits (NDUFS3, NDUFV2) and altered content of proteins involved in calcium homeostasis/signaling (calcineurin A, α1-syntrophin, annexin A4) in OGDM (n = 6) vs. ONGT (n = 6). Follow-up analyses showed reduced enzymatic activity of mitochondrial complexes C-I, C-III, and C-IV (−60–75%) in the OGDM (n = 8) compared with ONGT (n = 10) subjects, though no differences were observed for mitochondrial complex protein content. Upstream regulators of mitochondrial biogenesis and oxidative phosphorylation were not different between groups. However, AMPK phosphorylation was dramatically reduced by 75% in the OGDM women. These data suggest that GDM is associated with reduced skeletal muscle oxidative phosphorylation and disordered calcium homeostasis. These relationships deserve further attention as they may represent novel risk factors for development of GDM and may have implications on the effectiveness of physical activity interventions on both treatment strategies for GDM and for prevention of type 2 diabetes postpartum.

## Introduction

Gestational diabetes mellitus (GDM) is a rapidly growing public health concern. Adoption of new diagnostic criteria recommended by the American Diabetes Association (ADA) [1], [2] estimates a global prevalence of nearly one in five women (∼18%) who are considered at risk for GDM. Obesity occurs in ∼one in three women of child-bearing age [1], [3] and is a driving force accelerating the prevalence of GDM. GDM not only complicates pregnancy by increasing risk of pre-eclampsia and cesarean delivery, but is an independent risk factor for excess fetal growth and childhood obesity [3]–[7], and a consequence of even greater insulin resistance and nutrient availability than associated with maternal obesity alone [4]–[8]. In addition, GDM diagnosis identifies a population of women at markedly increased risk for future diabetes [8], [9], in part due to abnormal skeletal muscle signaling. Up to fifty percent of women diagnosed with GDM will go on to develop type 2 diabetes (T2DM) [9]–[11], and physical activity and dietary interventions to prevent this progression have been disappointing due to compliance difficulties [10]–[13]. Thus, understanding the pathogenesis of GDM is extremely important from a public health perspective for both maternal and child health.

Late in gestation, due to the demands of the placental-fetal unit and rapid depletion of glycogen stores, all women exhibit a shift in metabolism to increase reliance on lipid for metabolic substrate, a term called accelerated starvation. This metabolic shift is accompanied by a large decrease in skeletal muscle insulin sensitivity (−50%), both of which serve to allow for increased glucose supply to the growing fetus [12]–[14]. Women with GDM, however, demonstrate lower rates of whole body lipid oxidation both during early and late gestation [14], [15], with a less robust shift from glucose to lipid metabolism in late pregnancy compared with their normal glucose tolerant (NGT) counterparts [15]–[17]. Skeletal muscle, by virtue of its mass, is the principle site of glucose and lipid oxidation [3], [16]–[18], and therefore plays an important role in whether fuels are utilized by maternal tissues or shunted across the placenta to the developing fetus. The pathways underlying insulin resistance in GDM are well studied and multifactorial. However, little is known about the cellular mechanisms for altered skeletal muscle lipid or glucose metabolism, which is likely to significantly alter nutrient availability, thus increasing risk for increased fetal fat accretion and an increased risk of childhood obesity in offspring of women with GDM [3], [18], [19].

Therefore, the purpose of this study was to employ a discovery proteomic approach to identify candidate proteins that may underlie differences in skeletal muscle metabolism in obese, GDM pregnant women. The advantage of proteomic analysis, as opposed to a transcriptomic approach, is that we are able to measure content of global functional molecules involved in skeletal muscle metabolism rather than expression of genes that may or may not be related to protein content. We used an established quantitative proteomic analysis [19], [20] to compare skeletal muscle of obese pregnant women with GDM (OGDM) with obese pregnant women with NGT (ONGT). We then carried out a functional validation in a second, larger cohort of obese pregnant women with and without GDM. We are the first to demonstrate that several proteins of the mitochondrial electron transport system (ETS) are downregulated in the OGDM women, a factor we corroborated by demonstrating reduced activity of several enzyme complexes of the ETS in the second cohort of women. Proteomic analysis also revealed disruptions in calcium signaling/homeostasis proteins in skeletal muscle of the obese women with GDM, several of which may be related to cellular and mitochondrial stress. Along with a marked reduction in phosphorylated AMPK in the OGDM women, these results suggest that reduced capacity for skeletal muscle lipid or glucose oxidation may play an important role in the pathogenesis of GDM and may contribute to increased nutrient exposure to the fetus, potentially resulting in excess infant adiposity and long-term health consequences in the offspring.

## Research Design and Methods

### Study 1: Proteomic Analysis

#### Ethics Statement

Approval for this study was obtained from the East Carolina University Policy and Review Committee on Human Research and the Colorado Multiple Institutional Review Board at the University of Colorado Hospital. Written informed consent was obtained from all participants prior to cesarean delivery.

#### Patients and Sample Collection

Pregnant women over 21 years of age were screened for GDM between 24–28 weeks gestation and were diagnosed according to Carpenter and Coustan (CC) criteria [20], [21] using a 3-hour 100 g oral glucose tolerance test. Women with polycystic ovarian syndrome, pre-eclampsia, and macrovascular complications were excluded. All women with GDM were treated with insulin during the last 10–15 weeks of pregnancy to maintain normoglycemia. Body mass index (BMI) was calculated based on measurements from patients’ first antenatal visit (∼12 weeks gestation) and, in both groups, only those with a BMI of >30 kg/m^2^ were included. Plasma insulin and glucose were measured in samples obtained immediately prior to cesarean delivery.

Between 300–500 mg of rectus abdominus skeletal muscle tissue were obtained from a total of 12 obese pregnant women undergoing elective cesarean delivery (term, ∼37 weeks gestation; 6 ONGT women, 6 OGDM). Rectus abdominus is of mixed muscle fiber type [19], [21]. Dissections of skeletal muscle were obtained within 10 min of delivery, dissected free from visible adipose and connective tissues, and snap frozen in liquid nitrogen and stored at −80°C until further analysis.

#### Protein Isolation and Mass Spectrometry

Muscle protein content was determined as previously described [19], [22]. Approximately 30 mg of muscle tissue was homogenized in ice-cold lysis buffer as described [19], [22]. Tissue was homogenized while still frozen in an ice-cold buffer (10 µl/mg tissue) consisting of (final concentrations): 20 mM HEPES, pH 7.6; 1 mM EDTA; 250 mM sucrose, 2 mM Na_3_VO_4_; 10 mM NaF; 1 mM sodium pyrophosphate; 1 mM ammonium molybdate; 250 µM PMSF; 10 µg/ml leupeptin; and 10 µg/ml aprotinin. After homogenized by a polytron homogenizer on maximum speed for 30 sec, the homogenate was cooled on ice for 20 min and then centrifuged at 10,000×g for 20 min at 4°C; the resulting supernatant containing 60 µg of lysate supernatant proteins was used for in-gel digestion. Protein concentrations were determined by the method of Lowry method. Muscle lysate proteins were separated on 4–20% 1D linear gradient SDS polyacrylamide gels and each lane was cut into 20 separate slices. Gel pieces were treated with trypsin to digest proteins; the resulting mixture was desalted and subjected to mass spectrometry.

HPLC-ESI-MS/MS was performed on a hybrid linear ion trap (LTQ)-Fourier Transform Ion Cyclotron Resonance (FTICR) mass spectrometer (LTQ FT; Thermo Fisher, San Jose, CA) fitted with a PicoViewTM nanospray source (New Objective, Woburn, MA). On-line capillary HPLC was performed using a Michrom BioResources Paradigm MS4 micro HPLC (Auburn, CA) with a PicoFritTM column (New Objective; 75 µm i.d., packed with ProteoPepTM II C18 material, 300 Å). Samples were desalted using an on-line Nanotrap (Michrom BioResources, Auburn, CA) before being loaded onto the PicoFritTM column. HPLC separations were accomplished with a linear gradient of 2 to 27% ACN in 0.1% FA in 70 min, a hold of 5 min at 27% ACN, followed by a step to 50% ACN, hold 5 min and then a step to 80%, hold 5 min; flow rate, 300 nl/min. A “top-10” data-dependent tandem mass spectrometry approach was utilized to identify peptides in which a full scan spectrum (survey scan) was acquired followed by collision-induced dissociation (CID) mass spectra of the 10 most abundant ions in the survey scan. The survey scan was acquired using the FTICR mass analyzer in order to obtain high resolution and high mass accuracy data.

#### Data Analysis and Bioinformatics

Tandem mass spectra were extracted from Xcalibur “RAW” files and charge states were assigned using the Extract_MSN script (Thermo Fisher, San Jose, CA). The fragment mass spectra were then searched against the IPI_HUMAN_v3.59 database (80,128 entries, http://www.ebi.ac.uk/IPI/) using Mascot (Matrix Science, London, UK; version 2.2). The false discovery rate was determined by selecting the option to search the decoy randomized database. The search parameters used were: 10 ppm mass tolerance for precursor ion masses and 0.5 Da for production masses; digestion with trypsin; a maximum of two missed tryptic cleavages; fixed modification of carboamidomethylation; variable modifications of oxidation of methionine and phosphorylation of serine, threonine and tyrosine. Probability assessment of peptide assignments and protein identifications were made through use of Scaffold (version Scaffold_2_00_06, Proteome Software Inc., Portland, OR). Only peptides with ≥95% probability were considered. Proteins that contained identical peptides and could not be differentiated based on MS/MS analysis alone were grouped.

Protein and gene ontology annotation were performed as described, as were extraction of tandem mass spectra and verification of charge states and monoisotopic peak assignments [19]. To quantify protein abundance, normalized spectral abundance factors (NSAF)s were used [19]. Briefly, MS/MS spectra assigned to a protein were normalized to the number of amino acids for that protein, resulting in a spectral abundance factor (SAF). Each SAF was normalized against the sum of all SAFs in one sample, resulting in the NSAF value. This calculation is represented by the following equation, where *N* is equal to the number of proteins detected in a sample:
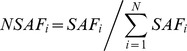
Thus, NSAF values allow for direct comparison of a protein’s abundance between individual runs in a fashion similar to microarray data analysis. The reproducibility and linearity of this method are described previously [19], [23].

### Study 2: Metabolic Analysis

#### Ethics Statement

Approval for this study was obtained from the Mercy Hospital for Women’s Research and Ethics Committee and written informed consent was obtained from all participants prior to cesarean delivery.

#### Patients and Sample Collection

To extend our observations from Study 1, a larger cohort of ONGT and OGDM subjects were studied. Pregnant women over 21 years of age were screened for GDM at 24–28 weeks gestation and were diagnosed according to the criteria set by the Australasian Diabetes in Pregnancy Society (ADIPS), by either a fasting venous plasma glucose level of 5.5 (compared to 5.3 using CC criteria) and/or greater than 8.0 mmol/L (8.6 using CC criteria) glucose 2 h after a 75 g oral glucose tolerance test. Applying these criteria results in a prevalence of GDM of ∼18% [23], [24]. Only GDM patients treated with insulin to control blood glucose levels were included. Women with polycystic ovarian syndrome, pre-eclampsia, and macrovascular complications were excluded. BMI was similarly calculated from patients’ first antenatal visit (∼12 weeks gestation) and only women in both groups with a BMI of >30 kg/m^2^ were included.

Between 300 and 500 mg of pyramidalis skeletal muscle was obtained from a total of 18 pregnant women undergoing elective caesarean delivery (term, ∼37 weeks gestation; 10 ONGT and 8 OGDM). The pyramidalis muscle is located anterior to the rectus abdominus and is also of mixed fiber type [24], [25]. Dissections of skeletal muscle were obtained within 10 min of delivery and snap frozen in liquid nitrogen and stored at −80°C until further analysis. Tissues were also imbedded for histology to verify that they were free from adipose or connective tissue contamination by hemotoxylin and eosin staining as previously described [25], [26].

For all deliveries in Study 2, spinal anesthesia and/or epidural were used. All muscle samples were taken at the time of cesarean delivery (between 0830 and 1500). Women delivering in the morning hours were fasted overnight, women delivering after 1200 were fasted from 0730.

#### Plasma Measures

Maternal blood was collected by venipuncture at the time of diagnosis (fasting sample, oral glucose tolerance test). Blood samples were immediately centrifuged at 1,500 *g* for 10 min and the plasma aliquoted into microfuge tubes and samples were immediately stored at −80°C until assayed for glucose and insulin. Blood glucose determination was performed by the hospital pathology department using an automated glucose oxidase/oxygen-rate method. Standard ELISA assay kit for insulin (Diagnostic Systems Laboratories, Webster, TX; limit of detection 0.26 IU/mL) was purchased and used according to the manufacturer's instructions. Insulin resistance at time of diagnosis was calculated using the homeostasis model assessment for insulin resistance (HOMA-IR) method where HOMA-IR = fasting plasma glucose (mmol/l) times fasting plasma insulin (µU/mL) divided by 22.5 [26], [27].

#### Mitochondrial Enzyme Activity Assays

Mitochondrial-enriched supernatants (post 600 g) were prepared from frozen skeletal muscle samples, as described [27]. Supernatants were used to assay activity of respiratory chain enzyme complexes I, II, III, and IV (C-I through C-IV, respectively); and citrate synthase (CS), spectrophotometrically on a Synergy H1 microplate reader (Biotek, Winooski, VT). Enzyme assays for respiratory chain complexes and CS were performed as described [27], [28] with minor modifications for microplate reading. For C-I and C-II, enzyme activities were calculated as initial rates (nmol/min). For complexes III and IV, enzyme activities were calculated as the first-order rate constants derived within 2–3 min of reaction initiation. All assays were performed in duplicate. The protein content of each sample was determined using a BCA assay. All activities were normalized to the total protein content and to CS activity (calculated as initial rate of reaction as nmol/min) of each sample and expressed relative to the mean for NGT women.

#### Western Blot

Protein levels of MitoProfile total OXPHOS antibody cocktail, AMPK, phosphoAMPK, PGC-1α and PPARα were determined in the muscle biopsy samples with calnexin as loading control as previously described [28]. OXPHOS antibody cocktail measured specific subunits of C-I, C-II, C-III, and C-IV (NDUFB8, SDHB, UQCRC2, and MT-CO1, respectively). All antibody dilutions were 1∶1,000 for primary antibodies and 1∶10,000 for secondary antibodies, unless otherwise stated. All results were expressed relative to the mean for NGT women. OXPHOS antibody cocktail (host: mouse), calnexin (host: rabbit), PGC-1α (host: rabbit), and PPARα (host: rabbit) antibodies were purchased from Abcam (Cambridge, MA). AMPK (host: rabbit) and phospho-AMPK (Thr172) (host: rabbit) antibodies were from Cell Signaling Technology (Danvers, MA). Secondary antibodies were purchased from Bio-Rad (Hercules, CA).

#### Mitochondrial DNA Copy Number

Approximately 15 mg of skeletal muscle was homogenized and DNA was isolated by phenol/chloroform extraction with ethanol precipitation. Mitochondrial DNA (mtDNA) copy number was then measured as previously described [28].

### Statistical Analyses

**Figure 1:**
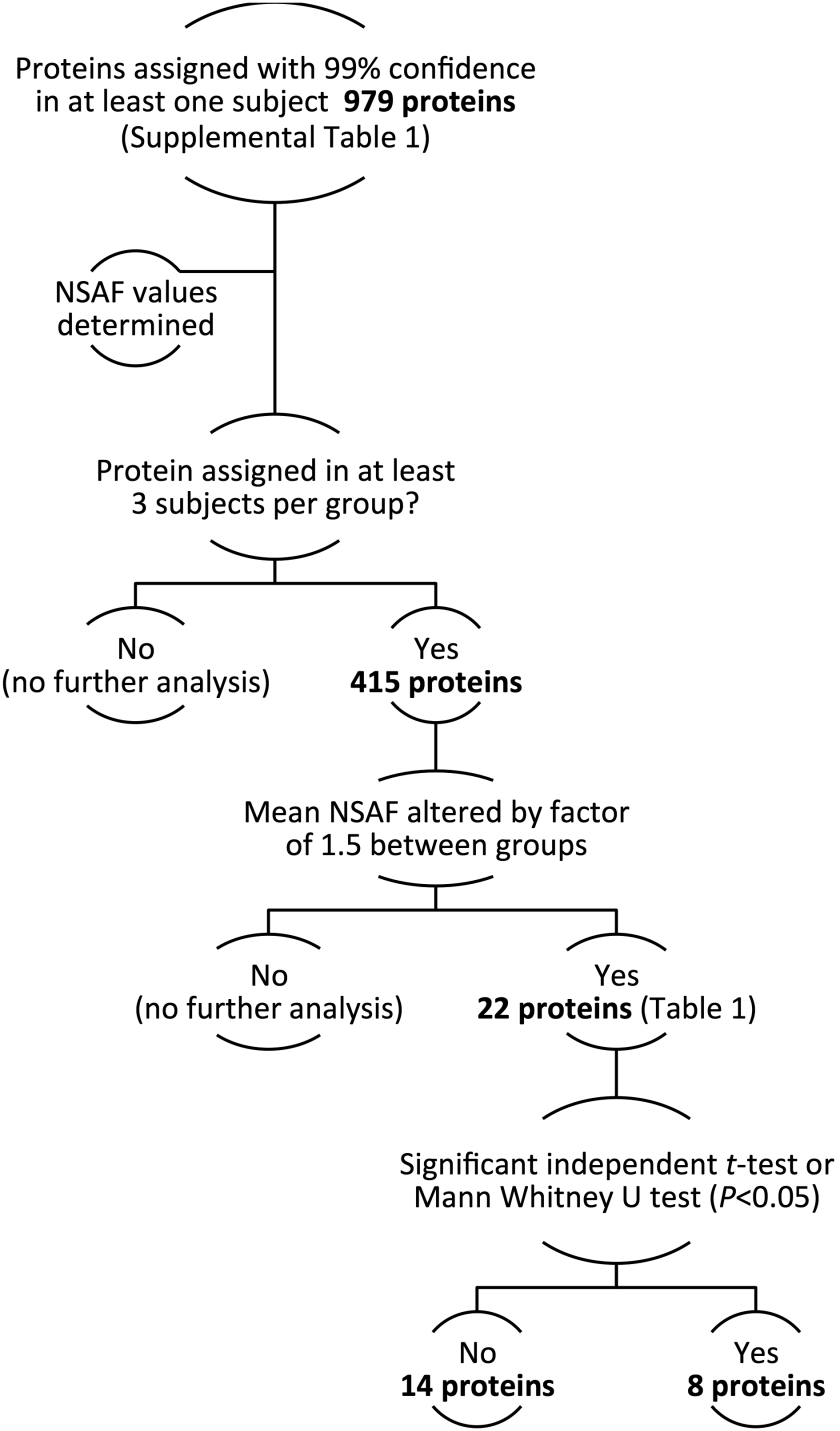
Work flow of statistical analysis of single proteins.

Statistical analyses were performed using IBM SPSS Statistics, Version 22 (IBM Corp., Armonk, NY). For the Study 1 proteomic analysis, a large number of proteins were assigned in at least one of 12 subjects studied (979 proteins; Table S1). A series of filters were used to narrow the number of proteins that were used in comparisons between groups (Figure 1). First only those proteins with representation in at least three subjects from each group were chosen (415 proteins). Of these, only those proteins with greater than 1.5-fold difference between groups (22 proteins) were examined for statistically significant differences between groups. First, data were tested for normality using the Shapiro-Wilk test, which were performed for ONGT and OGDM separately. Differences between groups were tested using non-parametric Mann Whitney U tests where data were not normally distributed, and remaining proteins were subjected to independent *t*-tests.

Study 2 was powered off our previous analysis of skeletal muscle enzyme activity in pregnant women [28]–[30], which showed a calculated effect size of 1.32 (averaged for C-I, C-II, and C-III) for differences between NGT and GDM women. We calculated 80% power with n = 10 subjects per group where α = 0.05 for two-sided independent *t*-tests. Data were tested for normality using the Shapiro-Wilk test, which were performed for ONGT and OGDM separately. Independent *t*-tests were performed on raw or log-transformed data where data were not normally distributed. Statistical difference is indicated by *P*≤0.05. Data are expressed as the mean ± SEM.

## Results

### Study 1: Proteomic Analysis

#### Patient Characteristics

Patient characteristics for the women in Study 1 are shown in Table 1. Women in the ONGT (n = 6) and OGDM (n = 6) groups were of similar BMI. As expected, OGDM women had higher fasting blood glucose and insulin values, which translated to higher HOMA-IR scores (*P<0.05*).

**Table 1:** Clinical characteristics of the subjects for Study 1.

	ONGT (n = 6)	OGDM (n = 6)
Delivery BMI (kg/m^2^)	34.2±2.3	37.5±2.1
Fasting Glucose (mmol/L)	4.2±0.2	5.3±0.3*
Insulin (µU/L)	11.3±3.2	19.5±4.8*
HOMA-IR	2.1±0.7	4.6±1.0*
Triglycerides (mg/dL)	161.0±22.6	265.5±40.3
Gravida	2.0±0.0	2.5±2.1
Parity	1.0±0.0	1.0±1.4

Data are mean ± SEM. OGTT, oral glucose tolerance test. Independent *t*-test *P<0.05.

#### Mitochondrial and Calcium Signaling Proteins are Altered in Women with GDM

Of the 979 unique proteins identified by tandem MS/MS mass spectrometry and NSAF analysis (Table S1), 22 met our criteria for statistical comparison between the OGDM and ONGT participants (protein detectable in at least three subjects per group and greater than 1.5-fold difference between groups; Table 2). Of these, eight proteins were statistically different between the groups (*P≤0.05*). Two of these proteins were subunits of ETS Complex I (C-I; NDUFV2 and NDUFS3), which were reduced by 60% in women with OGDM (*P<0.05*), while a third subunit of C-I (NDUFS8) tended to be reduced by 75% in the women with OGDM, compared with ONGT women (*P = 0.07*). Fatty acid transporter CD36 was increased by almost 75% in women with OGDM (P = 0.05). In addition, proteasome subunit alpha-1, a protein involved in protein degradation, was reduced by nearly 2-fold in women with OGDM (*P<0.05*). Surprisingly, several proteins involved in calcium homeostasis and calcium-dependent cellular signaling were also altered in skeletal muscle of women with OGDM, including calmodulin-dependent calcineurin A β (PPP3CB), which was increased by over 2-fold in women with OGDM compared with ONGT women (*P<0.05*). Similarly, calcium/calmodulin-dependent protein kinase type II, subunit A (CaMKIIa), tended to be higher in women with OGDM, though this was not significantly different between groups (+2-fold in GDM, *P = 0.15*). Furthermore, annexin A4, a calcium binding protein, was decreased by 65% in the women with OGDM (*P = 0.05*) and α1-syntrophin, a cellular localization protein involved in ion transport and cellular signaling, including calcium ion flux, was decreased by nearly 3-fold in women with OGDM (*P<0.05*).

**Table 2:** Proteins with 1.5-fold difference between ONGT and OGDM, Study 1.

Gene Name	Protein	Function	FoldDifference	*t*-test*P* value
MYOM1	Myomesin 1	Structural protein	5.21	0.07
HSPA1A/1B	Heat shock 70 kDaprotein 1A/1B	Stress response	2.98	0.09
ME1	NADP-dependentmalic enzyme	Pyruvate metabolism	2.39	0.09
CMBL	Carboxymethylenebutenolidasehomolog	Cysteine Hydrolase	2.30	0.01*
PPP3CB	Calmodulin-dependentcalcineurin A β	Calcium homeostasis	2.22	0.02*
CAMK2A	Calcium/calmodulin-dependentprotein kinase 2 α	Calcium homeostasis	2.17	0.15
FKBP3	Peptidyl-prolyl cis-transisomerase FKBP3	Protein folding	1.86	0.38
ERP29	Endoplasmic reticulumresident protein 29	Protein folding	1.75	0.40
CD36	Platelet glycoprotein 4	Fatty acid transport	1.73	0.05*
LRRC47	Leucine-rich repeat-containing protein 47	N/A	1.71	0.30
TUBA8	Tubulin alpha-8 chain	Cytoskeletonorganization	−1.54	0.10
CAPZA2	F-actin-cappingprotein subunit alpha-2	Cytoskeletonorganization	−1.58	0.12
NDUFV2	NADH dehydrogenaseflavoprotein 2	Oxidativephosphorylation	−1.59	0.04*
NDUFS3	NADH dehydrogenaseiron-sulfur protein 3	Oxidativephosphorylation	−1.60	0.03*
ANXA4	Annexin A4	Calciumbinding protein	−1.65	0.05*
NDUFS8	NADH dehydrogenaseiron-sulfur protein 8	Oxidativephosphorylation	−1.74	0.07
PSMA2	Proteasome subunitalpha type-1	Proteolysis	−1.97	0.04*
TTR	Transthyretin	Thyroid hormonetransport (circulation)	−1.97	0.10
SERPINC1	Antithrombin-III	Serine proteaseinhibitor (circulating)	−2.03	0.12
AHSG	Alpha-2-HS-glycoprotein	Calcium transport(circulating)	−2.07	0.07
CP	Ceruloplasmin	Iron transport(circulating)	−2.07	0.19
SNTA1	Alpha1-syntrophin	Ion transport(including calcium)	−2.93	0.03*

### Study 2: Metabolic Analysis

#### Patient Characteristics

For Study 2, we followed up the results from Study 1 in a larger, separate cohort of OGDM and ONGT women (Table 3). The ONGT (n = 10) tended to be more obese than the OGDM women (n = 8; P = 0.06), though fasting blood glucose and 1 and 2 hour OGTT glucose values were higher in OGDM than ONGT women (P<0.05).

**Table 3:** Clinical characteristics of the subjects for Study 2.

	ONGT (n = 10)	OGDM (n = 8)
Maternal age (years)	33.4±1.8	34.1±1.1
12-week BMI (kg/m^2^)	38.4±1.7	33.8±1.5
Delivery BMI (kg/m^2^)	41.2±2.2	36.8±1.6
Glucose: 0 h OGTT (mmol/L)	4.7±0.1	5.5±0.2*
Glucose: 1 h OGTT (mmol/L)	7.4±0.6	9.9±0.6*
Glucose: 2 h OGTT (mmol/L)	5.3±0.3	7.7±0.7*
Gestational age (weeks)	38.7±0.2	38,2±0.4
Gravida	3.5±0.6	3.0±0.3
Parity	2.4±0.4	2.0±0.3
Neonate birth weight (g)	3554.5±107.1	3616.1±183.0

Data are mean ± SEM. OGTT, oral glucose tolerance test. Independent *t*-test *P<0.05.

#### Mitochondrial Enzyme Activity is Reduced in Women with GDM

**Figure 2:**
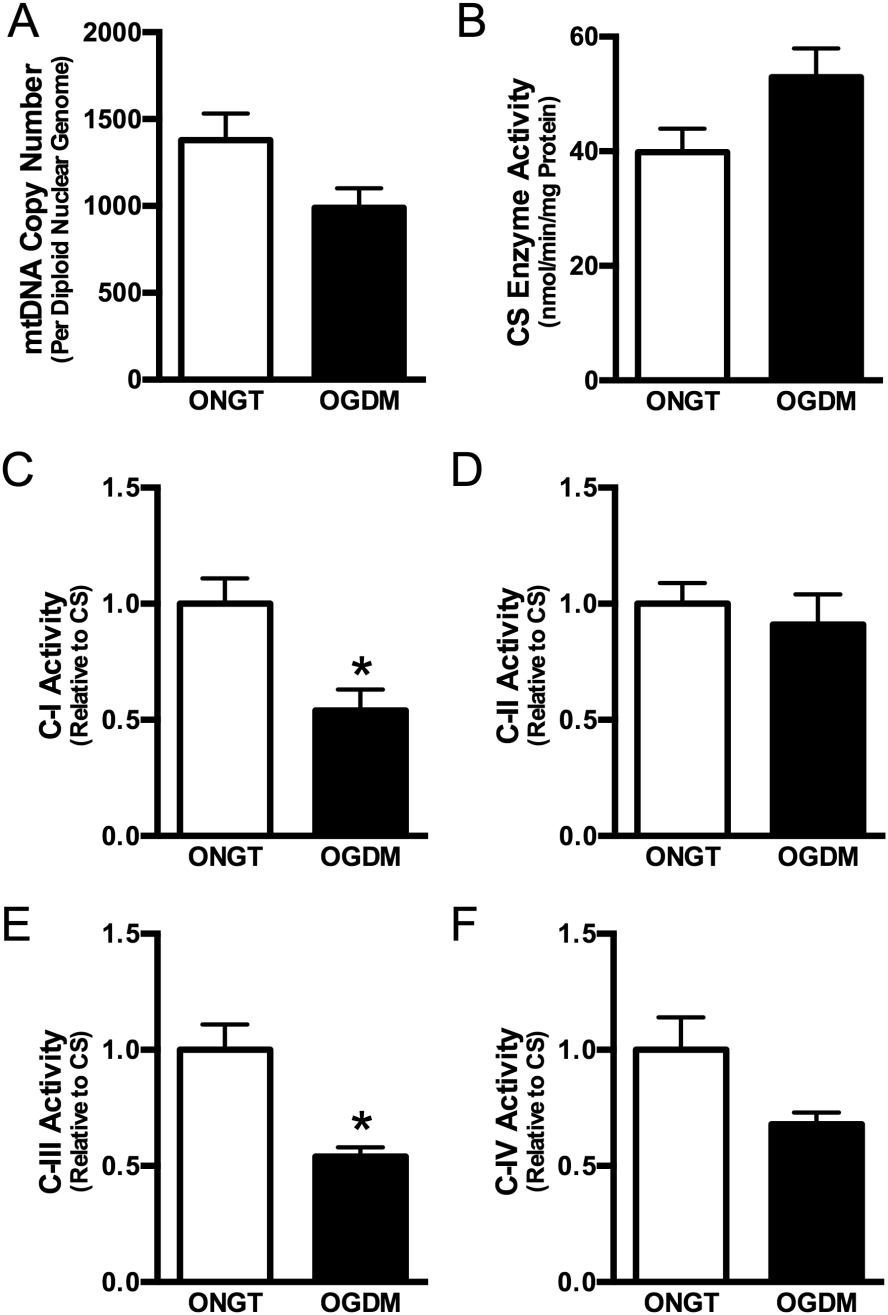
Mitochondrial enzyme activity is reduced in skeletal muscle of OGDM women. Quantitative bar graphs of mitochondrial DNA copy number (*A*) and enzyme activity of citrate synthase (*B*) complex I (*C*), complex II (*D*), complex III (*E*), and complex IV (*F*) of the respiratory chain in pyramidalis muscle collected during scheduled cesarean section. Data are mean ± SEM. *P<0.05 *vs*. NGT.

Because we observed reduced protein content of mitochondrial oxidative phosphorylation subunits (Study 1, Table 2), we measured two common markers of mitochondrial content (mitochondrial DNA copy number [mtDNA] and CS activity) in Study 2. However, mtDNA tended to be 30% lower in OGDM compared to ONGT women (*P = 0.10*; Figure 2*A*), while CS activity tended to be 30% higher in the women with OGDM (*P = 0.06*; Figure 2*B*).

**Figure 3:**
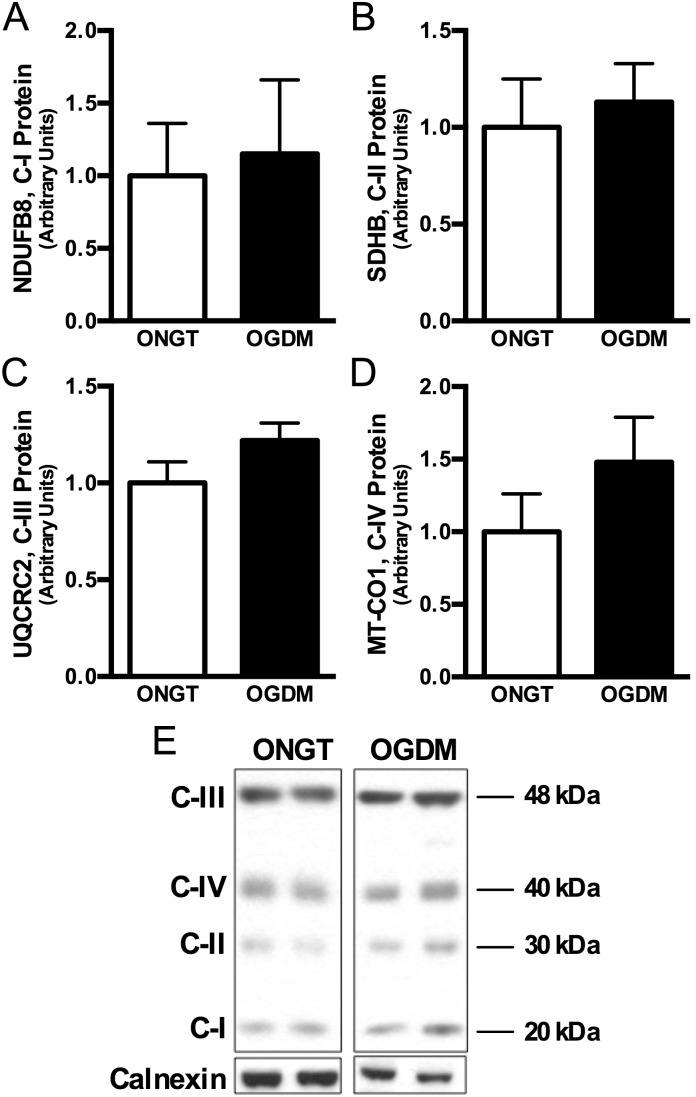
Mitochondrial respiratory chain complex proteins are not different between ONGT and OGDM women. Complex I (*A*), complex II (*B*), complex III (*C*), complex IV (*D*). Representative Western blots where calnexin is used as loading control (*E*).

Next, given the reduced content of several C-I subunits (NDUFV2, NDUFS3) in skeletal muscle of OGDM compared to ONGT women (Study 1), we determined whether the activity of key enzymes involved in mitochondrial oxidative phosphorylation was also different between groups in Study 2. We found that women OGDM women exhibit reduced activity of C-I and C-III, (by 45% and 45%, and 30%, respectively; Figure 2*C & E* *; P*<0.05) and a trend for reduced C-IV activity (by 30%; Figure 2F; *P* = 0.06) compared to ONGT women. However, OXPHOS protein content was not similarly reduced (Figure 3*A, C*, & *D*). There were no differences in C-II activity or protein content (Figure 2*D* & 3*B*).

**Figure 4:**
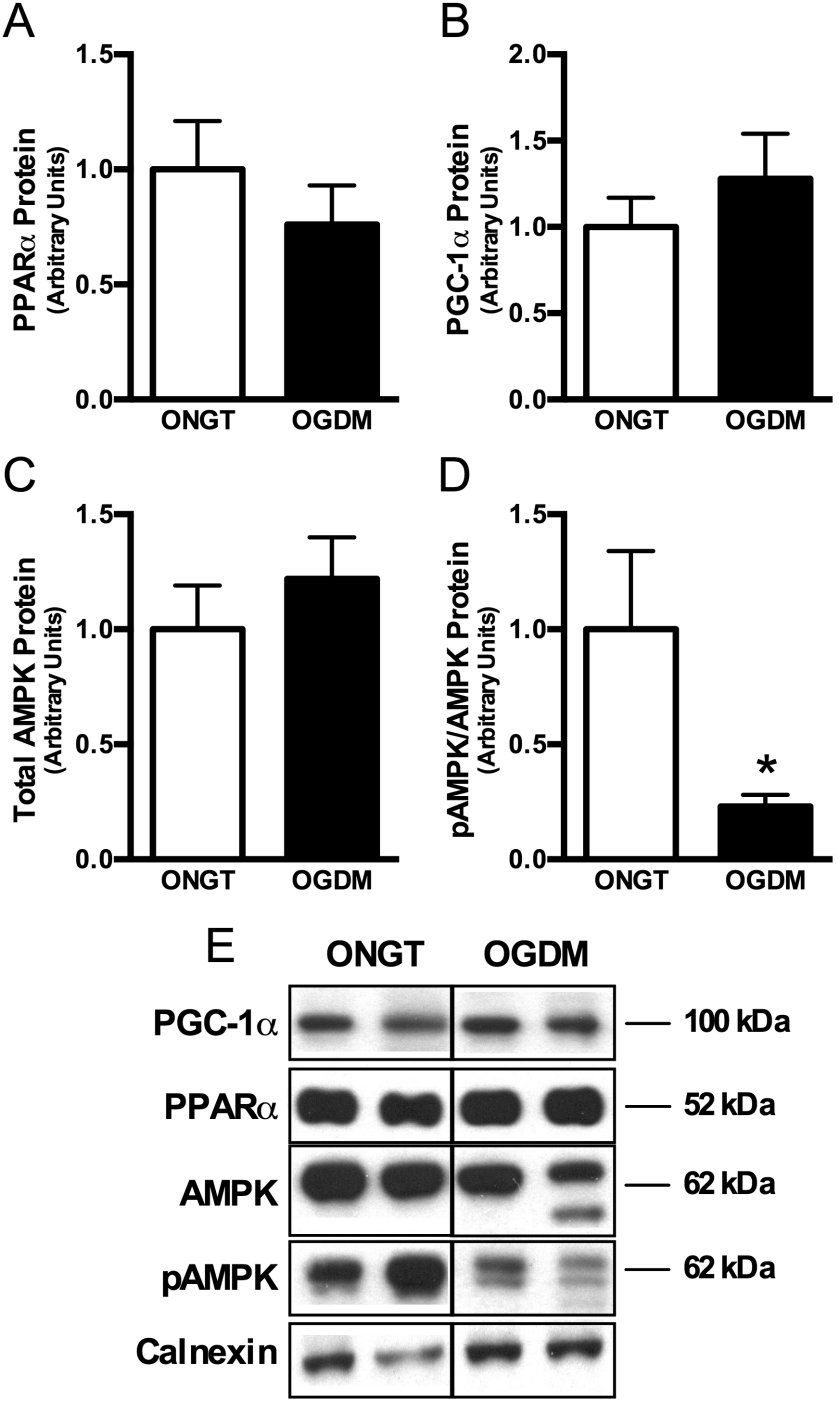
AMPK phosphorylation is reduced in OGDM women, though regulators of mitochondrial biogenesis are not. Quantitative bar graphs of PPARα (*A*) and PGC-1α (*B*), total AMPK (*C*), and phospho-AMPK (*D*) protein content in pyramidalis muscle collected during scheduled cesarean section. Representative Western blots where calnexin is used as loading control (*E*). Data are mean ± SEM. *P<0.05 *vs*. NGT.

To examine whether transcriptional regulators of mitochondrial biogenesis were affected, we measured PPARα and PGC-1α protein content, though these were not different between ONGT and OGDM women (Figure 4*A & B*; *ns*). Lastly, because AMPK is a calcium-dependent protein involved in cellular metabolism *and* mitochondrial biogenesis [29]–[33], we then measured AMPK phosphorylation and found that Thr172 phosphorylation of AMPK was markedly downregulated by 75% in OGDM compared to ONGT women (Figure 4*C & D*).

## Discussion

The present study is the first proteomic analysis of skeletal muscle tissue from obese, pregnant women with GDM compared with their obese, NGT counterparts. In this hypothesis-generating analysis we found that several proteins of the mitochondrial ETS were downregulated in the women with GDM, a factor that was corroborated by reduced activity of several enzyme complexes of the ETS in a similar, larger cohort of both OGDM and ONGT women. Proteomic analysis also revealed disrupted calcium signaling/homeostasis proteins in skeletal muscle of OGDM women. Subsequent measures also showed a pronounced reduction in the phosphorylation of AMPK in skeletal muscle in OGDM compared to ONGT women, which has been linked to both calcium homeostasis and reduced oxidative metabolism.

Obese, insulin resistant humans often exhibit reduced skeletal muscle mitochondrial function when compared with normal weight, insulin sensitive individuals, generally in the form of reduced electron transport flux or ATP turnover [31]–[34]. We found that obese women diagnosed with GDM showed significantly lower skeletal muscle mitochondrial C-I subunit protein content (Study 1) and enzyme activity (Study 2) compared to obese, NGT women. Even though there was a trend for reduced mtDNA copy number in the OGDM women, the trend for increased CS activity in these same women, makes it difficult to determine whether mitochondrial content was reduced in the women with GDM. However, given that enzyme activity data were normalized to CS activity in Study 2, the lower enzyme activities in the OGDM women do not appear to be the result of lower mitochondrial content and these discrepancies in mitochondrial content simply highlight the fact that these data are *markers* of mitochondrial content, not direct measures. For this reason we also measured upstream regulators of oxidative metabolism and mitochondrial biogenesis (PGC-1α and PPARα) and found that they were not different between the OGDM and ONGT women. These data indicate that mitochondrial functional capacity may be linked to specific subunit protein content rather than global mitochondrial deficits in the skeletal muscle of women with OGDM. Thus, in this cohort where the principal differences between the OGDM and ONGT women is impaired glucose tolerance, these results suggest that reduced mitochondrial enzyme capacity may be an important determinant of excessive glucose intolerance during pregnancy, perhaps contributing to postpartum diabetes risk. However, the association between reduced skeletal muscle mitochondrial capacity and T2DM is not universally reported. For example, Asian Indians with T2DM show greater skeletal muscle mitochondrial activity than their non-diabetic counterparts [34], [35], and Boushel et al. [35]–[37] report similar mitochondrial respiration rates in permeabilized muscle fibers from obese, type 2 diabetics compared with non-diabetic, overweight counterparts, suggesting that the relationship between T2DM and reduced mitochondrial capacity is not causal. Gestational diabetes, like T2DM, is characterized by both insulin resistance and pancreatic beta cell dysfunction [8], [36], [37]. Therefore, in addition to factors such as skeletal muscle mitochondrial dysfunction and skeletal muscle insulin signaling [8], [38]–[40], which can exacerbate insulin resistance, factors determining beta cell function likely play a prominent role in the development of GDM and subsequent T2DM postpartum.

Calcium homeostasis and calcium-dependent signaling play important roles in many skeletal muscle processes in addition to muscle contraction, such as insulin-mediated glucose uptake, AMPK signaling and mitochondrial biogenesis [38]–[41]. Our proteomic analysis revealed decreased α1-syntrophin and annexin A4, proteins involved in intracellular calcium signaling/homeostasis. In addition, we observed large increases in both calcineurin A and CaMKIIα in OGDM compared with ONGT women, though differences in CaMKIIα did not reach statistical significance. Such data suggest a disruption in calcium handling in skeletal muscle of women with GDM that warrants further investigation into its role in insulin signaling and mitochondrial capacity.

Alpha 1-syntrophin is a member of the syntrophin family of scaffold proteins that interacts with many signaling proteins, linking them with various cell surface receptors, ion channels, and downstream effectors [41]–[43]. In particular, α1-syntrophin, interacts with dystrophin and related proteins [38], [41]–[44] and disruption of the dystrophin-syntrophin complex has been suggested to promote the excessive calcium influx and altered MAPK and GTPase signaling observed in muscular dystrophy [38], [41], [44]–[46]. In this context, silencing of α1-syntrophin in myotube cultures induces cellular calcium influx via store-operated calcium channels [39], [40], [45], [46]. Thus, the observed reduction in α1-syntrophin in the skeletal muscle of OGDM women in our study may affect myocellular calcium levels. Contrary to transient increases in intramyocellular calcium that induce AMPK phosphorylation, such as occurs with muscle contraction, sustained elevations of intramyocellular calcium can *reduce* AICAR-induced AMPK phosphorylation in myotube cultures, which appears to be regulated by elevated CaMKII [29], [39], [40]. Thus, sustained elevations in cellular calcium may induce CaMKII to inhibit AMPK phosphorylation. As a potent activator of lipid oxidation and mitochondrial biogenesis [10], [11], [29], the reduced AMPK phosphorylation observed in Study 2 could explain their reduced mitochondrial enzyme capacity via calcium regulatory mechanisms. Though we did not observe differences in overall mitochondrial content in Study 2, as estimated by mtDNA copy number and citrate synthase activity, it is possible that there was a deficit in content of specific subunit proteins, which contributed to reduced enzyme activity. For example, the C-I molecule contains 45 subunit proteins [47]. Our proteomic analysis only identified 24 C-I subunit proteins assigned in at least 1 individual (Table S1), of which only two were statistically different between groups. This suggests that, while overall subunit content of C-I is not different in skeletal muscle of the OGDM women, content of specific, important C-I subunit proteins *is* reduced, resulting in reduced mitochondrial enzyme activity. Indeed, gene silencing of NDUFS3 and mutations in the NDUFV2 gene have been shown to lead to mitochondrial dysfunction, hypertrophic cardiomyopathy, and Leigh’s Syndrome [48], [49]. Thus, calcium regulatory control of AMPK phosphorylation and mitochondrial enzyme capacity may have clinical implications that may partially explain why physical activity interventions in women with GDM, for the control glycemia and prevention of its progression to T2DM, are disappointing and often associated with a high non-compliance rate [10], [11], [50]. While this is the first report of reduced α1-syntrophin in human insulin resistant skeletal muscle, similar observations have been made in diabetic Goto-Kakizaki rats, where skeletal muscle dystrophin and α1-syntrophin content was markedly reduced and thought to be a contributor to the excessive insulin resistance observed in these animals [50], [51].

Annexin A4 is a lipid-binding protein involved in exocytosis and ion transport in a calcium-dependent manner [51], [52]. Among its many functions, annexin A4 interacts with the p50 subunit of NF-kB, reducing is transcriptional activity [8], [52], [53]. Thus, the inflammatory cascade may be enhanced in skeletal muscle where annexin A4 protein is reduced. This may be one mechanism whereby inflammation is elevated in skeletal muscle of women with GDM, as we have shown previously [8], [53]. As a result, serine phosphorylation of JNK and/or p70S6 kinase may link inflammation with disrupted insulin signaling in skeletal muscle of women with GDM [8], [53], [54]. While this is the first report of reduced annexin A4 in skeletal muscle of GDM patients, members of our group have recently reported reduced content of other annexin proteins (A2, A5, A6) in placental tissue from obese GDM patients [54], [55], suggesting a universal disruption of annexin protein content in pregnant women with GDM.

Calcineurin A was reduced in skeletal muscle of OGDM women. Calcineurin A is the catalytic subunit of calcineurin that forms a heterodimer with the calcineurin B regulatory subunit [55], [56]. Once activated by calcium/calmodulin, calcineurin stimulates insulin sensitizing pathways and has also been shown to induce lipid oxidation and mitochondrial biogenesis pathways, including increased expression of CD36 and all of the ETS complexes [56], [57], which may explain increased CD36 protein in OGDM women in the proteomic analysis. Increased calcineurin A may also account for the slight, yet non-significant increases in electron transport complex proteins measured in the OGDM women (Study 2). However, studies show that calcineurin expression is induced in response to mitochondrial stress and mtDNA damage [57], [58]. For example, in early stage diabetic cardiomyopathy, mitochondrial stress is commonly observed, along with disrupted calcium homeostasis, elevated calcineurin, and insulin resistance [28], [58]. While these observations may seem paradoxical given calcineurin action, they seem to suggest a compensatory function of calcineurin, with regard to mitochondrial biogenesis and insulin sensitivity, which may be precluded in cardiac muscle of diabetic cardiomyopathy patients. Perhaps a similar compensatory increase in calcium is observed in skeletal muscle of GDM patients in response to mitochondrial stress.

We have previously reported that obese women with mild, diet-treated GDM have similar skeletal muscle mitochondrial enzyme activity to obese NGT pregnant women [28], [59], [60]. Here, we have found that those with more severe GDM (insulin-treated) exhibit lower mitochondrial enzyme activity for C-I and C-III than obese NGT pregnant women, despite the tendency for lower BMI, suggesting that the extent of glucose intolerance may play a more important role in skeletal muscle mitochondrial enzyme capacity than BMI in these women. This is further corroborated by the fact that the women with insulin treated GDM presented here had similar BMIs as the women with diet-treated GDM reported previously [28]. While it is possible that exogenous insulin administration may have induced the observed proteomic or enzymatic differences in the OGDM women, it is equally plausible that, due to skeletal muscle insulin resistance of the OGDM women, exogenous insulin simply equalized the effect of insulin on skeletal muscle tissues to those observed in the non-insulin treated women. Nevertheless, reduced mitochondrial enzyme activity in the OGDM women does not appear to be the result of differences in total mitochondrial content, but rather may be the result of reduced subunit protein content. Even though we discovered reduced content of two C-I subunits, and a trend for reduced content of a third C-I subunit in our proteomic analysis, we did not observe differences in total OXPHOS content, which is likely due to the fact that the OXPHOS antibody cocktail only measures the NDUFB8 C-I subunit. Nevertheless, we must recognize that these observations can only be extended to obese women diagnosed with GDM, and may differ for those women with less severe GDM (diet-treated).

GDM women and their offspring are a population which globally and significantly contributes to the escalation of T2DM and childhood obesity and our proteomic analysis of skeletal muscle from obese pregnant women revealed that proteins involved in mitochondrial oxidative phosphorylation are reduced in OGDM compared to their ONGT counterparts. These observations were corroborated by reduced skeletal muscle mitochondrial enzyme activity in a larger cohort of obese pregnant women. We further demonstrated that several proteins involved in calcium homeostasis/signaling were altered in obese women with GDM, several of which may be linked to disrupted insulin signaling and reduced mitochondrial activity. Such disturbances in calcium homeostasis proteins could contribute to the inflammation, insulin resistance, and altered substrate metabolism observed in skeletal muscle of OGDM patients, consequently shunting maternal nutrients to the growing fetus. Furthermore, disruption of calcium homeostasis could help explain the high failure rate of exercise intervention studies designed to treat women with GDM during pregnancy and/or to prevent the development of T2DM postpartum [59], [60].

In conclusion, this exploratory analysis identifies several calcium regulatory proteins that could represent novel risk factors for both the development of GDM and the progression to T2DM. Larger, more targeted studies are required to confirm these exploratory analyses. Further examination of calcium regulatory proteins could reveal an important link between insulin resistance and reduced mitochondrial activity in obese women with GDM that could potentially be targeted for strategies geared toward prevention and treatment of T2DM in this rapidly growing cohort of at risk women.

## Supporting Information

Table S1 **Spectral counts and NSAF values for 979 proteins identified in at least 1 subject.**
(XLSX)
